# The Top-Cited Original Articles on the Role of Microglia in Neurodegenerative Diseases: A Bibliometric and Visualized Study

**DOI:** 10.3389/fnagi.2022.869964

**Published:** 2022-04-05

**Authors:** Mengjiao Sun, Xiaoling Li, Jing Sun, Hongxia Wang, Qinfang Xie, Manxia Wang

**Affiliations:** Department of Neurology, Lanzhou University Second Hospital, Lanzhou, China

**Keywords:** microglia, neurodegenerative diseases, bibliometric analysis, top 100 cited articles, visualized study

## Abstract

**Background:**

Microglia participants to neuronal loss during brain development, inflammation, ischemia, and neurodegeneration. This bibliometric and visualized study aimed to confirm the top 100 cited original research in the field and to analyze their characteristics.

**Methods:**

The Web of Science database (WOS) was retrieved using the specific search strategy. The top 100 cited original articles that focused on the role of microglia in neurodegenerative diseases (NDs) were filtered by two researchers independently. The trend of yearly publications and citations, citation densities, languages, and global contributions were analyzed. The highly cited countries, authors, institutions, and journals were visualized by bibliographic coupling analysis. The highly cited authors and journals in the references were visualized by co-citation analysis. The research hotspots were revealed by co-occurrence analysis and burst detection of author keywords.

**Results:**

The top 100 cited articles were published during the period 1988 to 2019. The peak of publication occurred in 2005 and 2006. The yearly total citations presented a rising trend. The highly cited articles were contributed by 26 countries, the United States was the country with the overwhelming number of publications and cited times. Stevens, Beth was the author with the largest number of cited times. Mcgeer PL was the author most frequently cited in the references. Harvard University was the institution with the greatest number of cited times and publications. Nature was the journal with the largest number of cited times. Journal of neuroscience was both the most often published and most frequently cited journal in the references. “Microglia”, “inflammation”, “Alzheimer’s disease” were the most frequently used keywords, and their average occurred time was around 2005. “Dementia,” “delirium,” “priming” were keywords that averagely occurred around 2010. The burst detection revealed that “TNF-beta,” “macrophage,” and “inflammation” were keywords that frequently burst in recent years.

**Conclusion:**

This bibliometric and visualized study revealed the top 100 cited original research that discussed the role of microglia in NDs. The United States was the biggest contributor, Harford University was the most influential institution. Journal of Neuroscience was the most often published and cited journal. Alzheimer’s disease was the hotspot in microglia and NDs. Recent research mainly focused on inflammation.

## Introduction

Neurodegenerative diseases (NDs) are heterogeneous diseases that are closely related to aging, the chronic progressive neuronal death eventually leads to the loss of structure and function in specific brain regions (Ellis and Fell, 2017; Jellinger, 2010; Kim et al., 2015). With the trend of global population aging, the number of patients with NDs like Alzheimer’s disease and Parkinson’s disease is rising yearly (Behl, 2021; Manes, 2016; Tzvetkov and Atanasov, 2018). In addition, the incidence of multiple sclerosis, stroke-induced secondary neurodegeneration, Huntington’s disease, human immunodeficiency virus-related neurocognitive impairment, Creutzfeldt-Jakob disease, amyotrophic lateral sclerosis, and are also growing yearly, bringing huge economic burden (Yeung et al., 2021).

Microglia are considered to be the main driving cells leading to neuronal damage in NDs (Song and Suk, 2017; Spittau, 2017). Overactivation and imbalance of microglia may lead to catastrophic and progressive neurotoxic reactions (Kim and Joh, 2006; Mcgeer et al., 2006; Zecca et al., 2006). However, the mechanism of neurotoxic activation of microglia remains unknown. In addition, microglia will continue to be activated under the stimulation of harmful factors for a long time (Španić et al., 2019). Furthermore, whether microglia phagocytosis is beneficial or harmful remains controversial, most researchers tend to support the beneficial role (Weldon et al., 1998; Ito et al., 2007; Sierra et al., 2010).

Although the role of microglia in NDs is still elusive, it was found that microglia participate in not only the development and maintenance of central nervous system homeostasis but also in the progress of NDs, especially in Alzheimer’s disease. It is of great significance to investigate the interaction between microglia and neurons and the specific mechanism of microglia in the regulation of neural circuits (Bisht et al., 2016; Delpech et al., 2016; Matcovitch-Natan et al., 2016; Nelson et al., 2017), thus it is necessary to find the most influential original research in this field.

By designing this bibliometric and visualized study, we retrieved and screened the highly cited original articles that focused on the role of microglia in NDs in the past 40 years, a bibliometric and visualized study was performed to reveal the trends of publications and citations, highly cited authors, institutions, countries, journals, as well as the hotspots in this field.

## Materials and Methods

### Retrieve of Literature

On February 21, 2022 (The literature were re-retrieved and the newest citation data were used during revision as reviewers suggested), we conducted a literature search based on the Web of Science database (WOS), including the Web of Science Core Collection database, BIOSIS Previews, KCI-Korean Journal Database, MEDLINE, Russian Science Citation Index, and SciELO Citation Index. The following strategy was used in the advanced search: TS = Microglia* AND TS = (Neurodegenerative Diseases* OR Alzheimer’s Disease* OR Parkinson’s Disease* OR Multiple Sclerosis* OR Huntington’s Disease* OR Amyotrophic Lateral Sclerosis*). The range of publication dates was set from January 1, 1980, to December 31, 2021, without the restriction of languages.

### Literature Screening

The literature screening was performed by two authors independently (Sun MJ and Li XL) to reveal the highly cited research in the field. Briefly, the search results were ranked by citations, and a threshold of citations was set as a cutoff value. The following inclusion criteria were used: (1) Original articles, either experimental or clinical research. (2) The main topic focused on the role of microglia in NDs, as listed in the search strategy, either the microglia-related pathogenesis, mechanism, biomarker, or pathological features. (3) Considering the cited times of the recently published articles were less than that of articles published earlier, articles that ranked at the end of the list but were published within 5 years (2017–2021) were included. The selected articles were added to the “marked list” on the WOS Website for further analysis.

### Data Extraction

The publication and citation data were analyzed online on the WOS Website using the “Citation Report” function. Including Times Cited and Publications Over Time, citing articles, and H-index. The contributions of countries, authors, and institutions to publications were evaluated by calculating the record counts, in the other words, how many times do they appeared in the author list. The citation density was defined as the citations per year since published (total citations/literature age). The language was analyzed online on the WOS Website using the “Citation Report” function. The top 10 cited in each field were listed to reveal the most productive and influential contributors and publishers. To emphasize the clinical guiding significance, the top 10 cited reviews in this field were screened and analyzed in the discussion.

### Visualized Citation Analysis

The top 100 most cited articles were exported from the WOS website, the visualized citation analysis was performed by using the Vosviewer (1.6.18, Leiden University, Netherlands). Visualized networks based on bibliographic data were presented, including the full counting bibliographic coupling analysis of countries, authors, institutions, journals. Co-citation analysis of cited authors and cited journals in the references.

### Research Hotspots

The research hotspots were revealed by analyzing the author keywords, including the frequently occurred keywords and the timeline of appeared years, which were analyzed in the Vosviewer by using the co-occurrence analysis. Visualized networks of frequently occurred keywords and the timeline of keywords ranked by average appeared years was made. The burst test of keywords was used to reveal the keywords that frequently appeared in some period, which was performed by using the Citespace software (version 5.8.R3, Drexel University, United States), a burst map of author keywords was made.

## Results

### Top 100 Cited Articles

**FIGURE 1:**
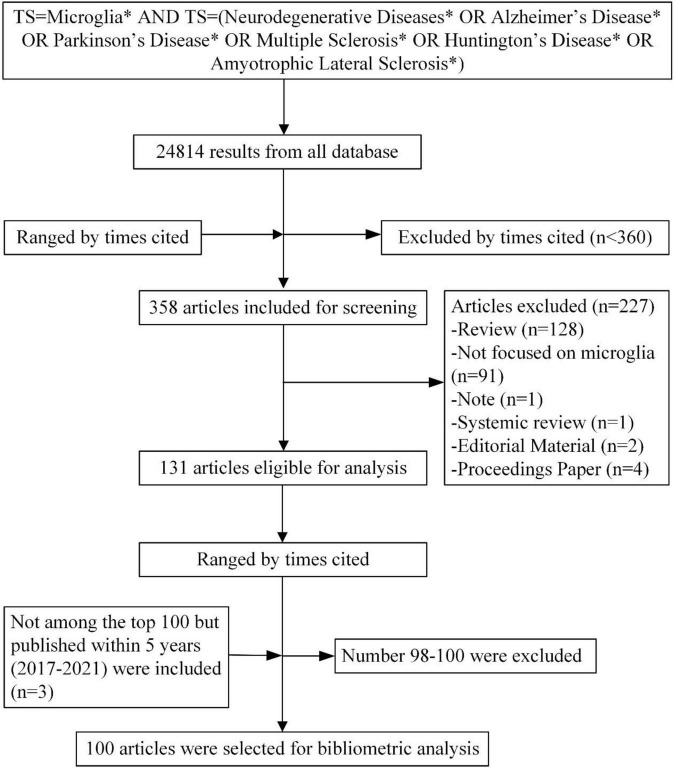
Flowchart of the literature search and article screening. (* is a wildcard character).

By the time we retrieved, the search strategy returned 24,814 results from all databases. After ranking by times cited, a minimal threshold of 360 cited times was set as a cut-off value, 358 articles fulfilled the requirements. The independent screening excluded 227 articles (including 128 reviews, 91 articles that were not focused on microglia and NDs, 1 Note, 1 systemic review, 2 editorial materials, and 4 proceedings papers). Among the 131 left articles, the top 100 articles with the highest cited times were chosen for bibliometric analysis, including 3 articles that were not among the top 100 but were published within 5 years (Figure 1). The full list of the top 100 most cited articles was presented in Supplementary Table 1.

### Publications and Citations

**FIGURE 2:**
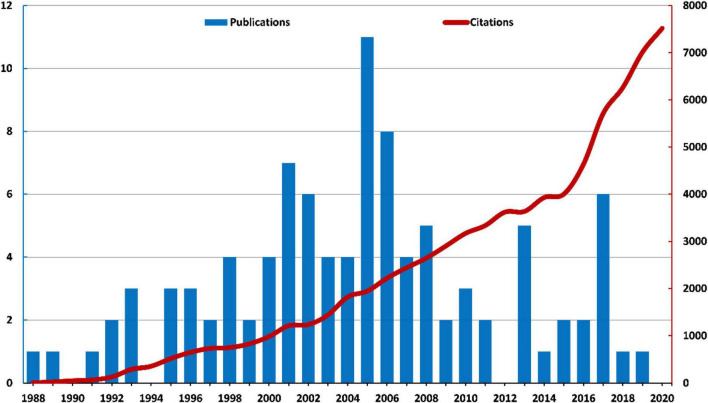
Trend of yearly publications and citations.

**FIGURE 3:**
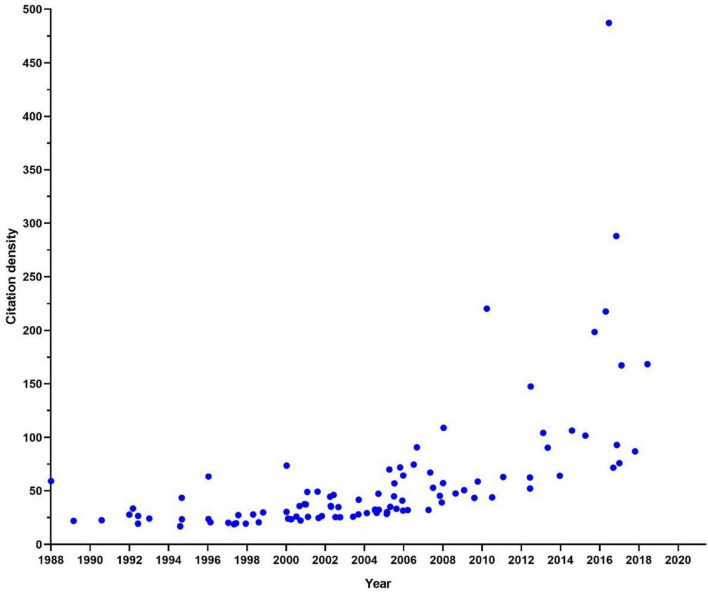
Scatter plot of citation densities of each article.

The top 100 highly cited articles were all from the WOS Core Collection and published between 1988 and 2019, all were published in English. The total H-index was 100. The peak in the number of publications occurred in the year 2005 and 2006, with 11 and 8 articles, respectively. The number of cited times of each article ranged from 348 to 2643. The top 100 highly cited articles were cited for a total of 76,679 times at the time of analysis, the number was 76,482 without self-citations. These articles cited 50,087 articles in their references, the number was 50,013 without self-citation. The yearly citations from 1988 to 2020 presented a rising trend (Figure 2). There was a weak rising trend in the citation densities since 2010, articles with high citation densities frequently occurred between 2010 and 2019. The majority of articles had less than 100 citation densities (Figure 3).

### Countries

**FIGURE 4:**
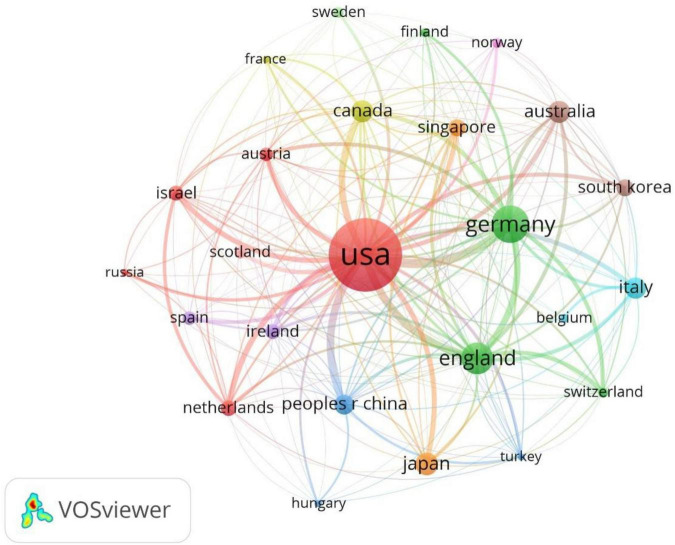
Bibliographic coupling analysis highly cited countries, weighted by citations, visualized map.

**FIGURE 5:**
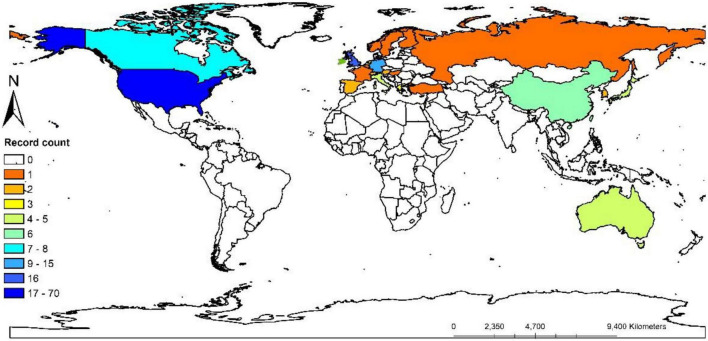
Global distribution record counts of highly cited articles.

The top 100 highly cited articles were from 26 countries (Figure 4), when ranked by cited times, the United States was the country with the largest number of cited times and record counts, with 56,122 total cited times and 70 record counts, the average cited times per item was 801.74. The second large contributor was Germany, with 14,368 total cited times and 15 record counts, the average cited time per item was 957.87. The third was England, with 10,460 total cited times and 14 record counts, the average cited time per item was 747.14. The global record counts of the top 100 highly cited articles were presented in the world map (Figure 5). The top 10 countries with the greatest number of cited times were listed in Table 1.

**TABLE 1:** List of the top 10 cited countries.

Country	Total cited times	Record count	Citations per item
United States	56,122	70	801.74
Germany	14,368	15	957.87
England	10,460	14	747.14
Japan	5,296	5	1,059.2
Canada	5,101	8	637.63
Australia	4,905	5	981
Italy	4,638	5	927.6
China	4,174	6	695.67
Singapore	3,148	2	1,574
South Korea	2,956	2	1,478

### Authors

**FIGURE 6:**
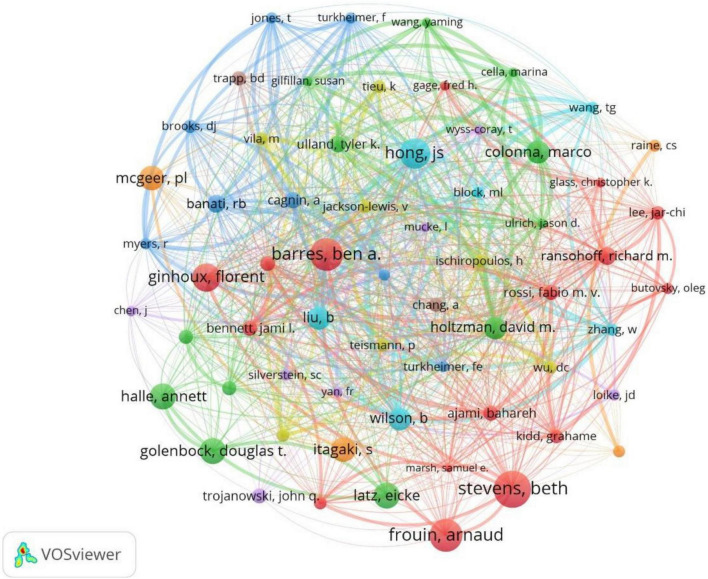
Bibliographic coupling analysis highly cited authors, weighted by citations, visualized map.

The full counting bibliographic coupling analysis of authors revealed the highly cited authors (Figure 6). When ranked by citations, the top author with the highest cited times was Stevens, Beth, with 4,131 cited times and three record counts. Followed by Barrens Ben A. and Frouin Arnaud, both were cited by 3,626 times had two record counts. The top 10 authors with the largest numbers of cited times were listed in Table 2.

**TABLE 2:** List of the top 10 cited authors.

Author	Affiliation listed in articles	Times cited	Record count	Citations per item
Stevens B	Boston Children’s Hospital	4,131	3	1,377
Barrens BA	Stanford University	3,626	2	1,813
Frouin A	Boston Children’s Hospital	3,626	2	1,813
Hong JS	National Institute of Environmental Health Sciences	3,337	5	667.4
Ginhoux F	Icahn School of Medicine at Mount Sinai	3,148	2	1,574
Golenbock DT	University of Massachusetts	2,852	2	1,426
Halle A	University of Massachusetts	2,852	2	1,426
Latz E	University of Bonn	2,852	2	1,426
Itagaki S	University of British Columbia	2,732	2	1,366
Mcgeer PL	University of British Columbia	2,732	2	1,366

**FIGURE 7:**
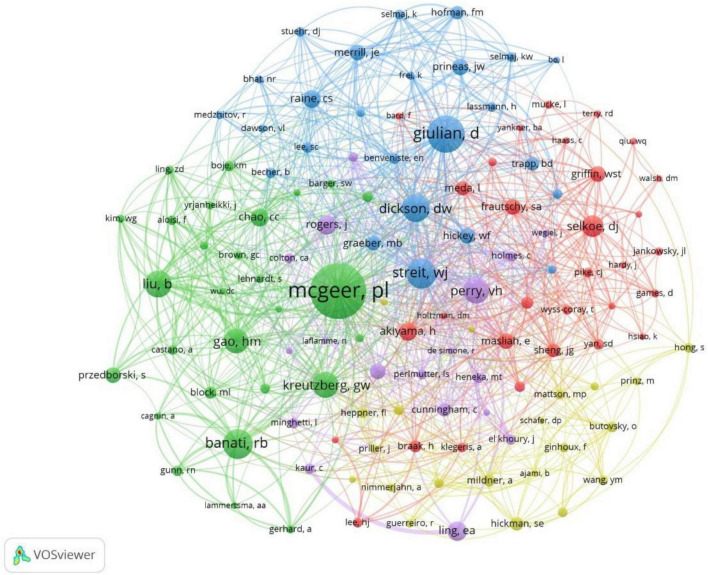
Co-citation analysis of most frequently cited authors in the reference lists, weighed by citations, visualized map.

The full counting co-citation analysis of cited authors revealed authors who were most frequently cited in the references (Figure 7). When ranked by citations, the top-cited author was Mcgeer PL (cited by 50 times), Giulian D (cited by 32 times), Streit WJ (cited by 25 times), Banati RB (cited by 25 times), Dickson DW (cited by 23 times). The top 10 cited authors in the references was listed in Table 3.

**TABLE 3:** List of the top 10 cited authors in the references.

Author	Affiliation listed in articles	Times cited	Total link strength
Mcgeer PL	University of British Columbia	50	344
Giulian D	Baylor College of Medicine	32	178
Streit WJ	University of Florida	25	222
Banati RB	Imperial College School of Medicine	25	122
Dickson DW	Albert Einstein College of Medicine	23	244
Liu B	National Institute of Environmental Health Sciences	22	177
Perry VH	University of Southampton	21	130
Kreutzberg GW	Max-Planck Institute of Psychiatry	21	118
Gao HM	National Institute of Environmental Health Sciences	20	141
Selkoe DJ	Brigham & Women’s Hospital	17	68

### Institutions

**FIGURE 8:**
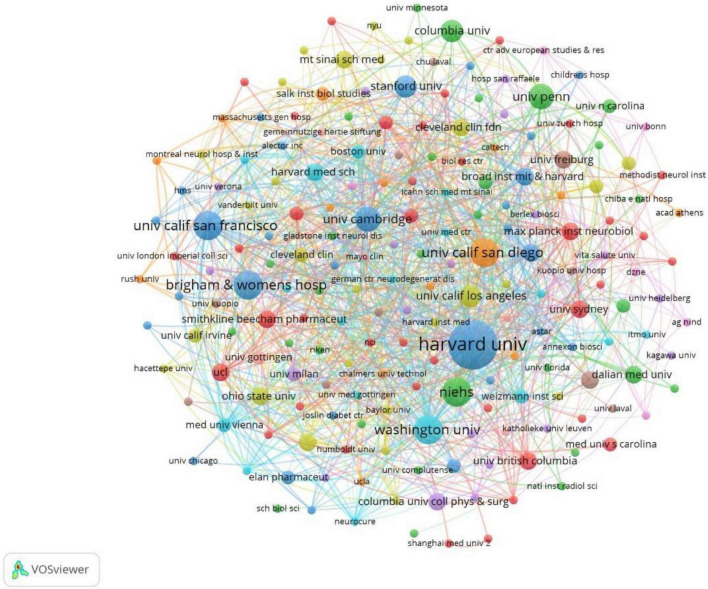
Bibliographic coupling analysis highly cited institutions, weighted by citations, visualized map.

The full counting bibliographic coupling analysis of institutions revealed highly cited institutions when weighed by citations, the top 100 cited articles addressed 193 institutions (Figure 8). The institution with the largest number of cited times was Harvard University, with 10,370 citations and 13 record counts. Followed by the University of California San Francisco, with 6,091 citations and 6 record counts. The third was Stanford University, with 5,176 citations and 4 record counts. The top 10 institutions with the greatest number of cited times were listed in Table 4.

**TABLE 4:** List of the top 10 cited institutions.

Institution	Country	Times cited	Record count	Citations per item
Harvard University	United States	10,370	13	797.69
University of California San Francisco	United States	6,091	6	1,015.17
Stanford University	United States	5,176	4	1,294
University of Cambridge	England	4,933	5	986.6
Washington University Wustl	United States	4,774	6	795.67
University of California San Diego	United States	4,528	6	754.67
National Institute of Environmental Health Sciences	United States	4,367	6	727.83
Brigham Women S Hospital	United States	4,245	6	707.5
Icahn School of Medicine at Mount Sinai	United States	4,204	3	1,401.33
Boston Children S Hospital	United States	4,131	3	1,377

### Journals

**FIGURE 9:**
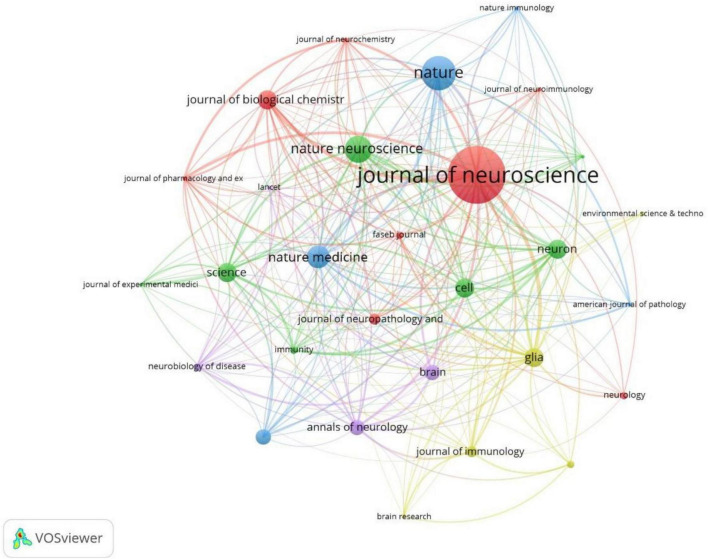
Bibliographic coupling analysis highly cited journals, weighted by citations, visualized map.

The full counting bibliographic coupling analysis of journals revealed that the top 100 highly cited articles were concentrated published in 29 journals (Figure 9). When ranked by citations, the top journal with the largest number of citations was Nature, with 9,675 citations and 9 publications. The second journal with the high citations was the Journal of neuroscience, with 9,137 citations and 15 publications. The third was nature neuroscience, with 5,982 citations and 7 publications. The top 10 journals that were most cited were listed in Table 5.

**TABLE 5:** List of the top 10 cited journals.

Journal	Times cited	Publications	Citations per item	Impact factor (2020)
Nature	9,675	9	1,075	49.962
Journal of Neuroscience	9,137	15	621.13	6.617
Nature Neuroscience	5,982	7	854.57	24.884
Science	5,931	5	1,186.2	47.728
Cell	4,489	5	897.8	41.584
Nature Medicine	4,262	6	710	53.44
Glia	3,862	5	772.4	7.452
Neuron	3,560	5	712	17.173
Annals of Neurology	2,959	4	739.75	10.422
Brain	2,813	4	703.25	13.501

**FIGURE 10:**
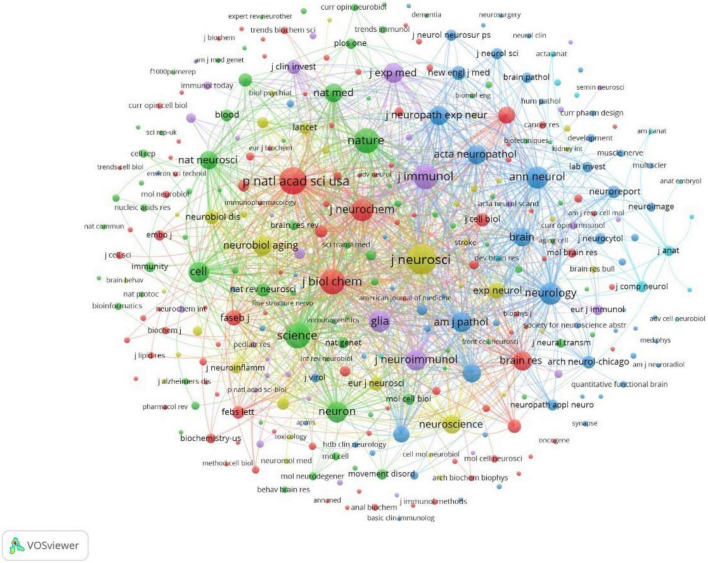
Co-citation analysis of most frequently cited journals in the reference lists, weighed by citations, visualized map.

The full counting co-citation analysis of cited journals showed that journals most frequently cited in the references were Journal of neuroscience (cited by 274 times), PNAS (cited by 206 times), Science (cited by 178 times), Nature (cited by 164 times) (Figure 10). The top 10 cited journals in the references were listed in Table 6.

**TABLE 6:** List of the top 10 cited journals in the references.

Journal	Times cited	Total link strength	Impact Factor (2020)
Journal of Neuroscience	274	10,995	6.617
PNAS	206	7,576	9.412
Science	178	6,167	47.728
Nature	164	5,887	49.962
Journal of biological chemistry	159	5,642	5.157
Journal of Immunology	159	5,187	5.422
Annals of Neurology	118	4,705	10.422
Neurology	118	4,629	9.91
Journal of Neurochemistry	113	4,493	5.372
Glia	106	3,999	7.452

### Hotspots

**FIGURE 11:**
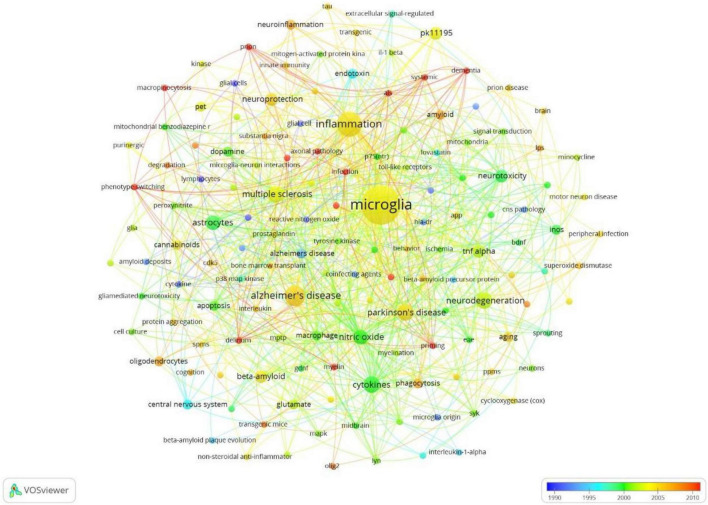
Co-occurrence analysis of frequently occurred author keywords, weighed by occurrence, visualized timeline map.

**FIGURE 12:**
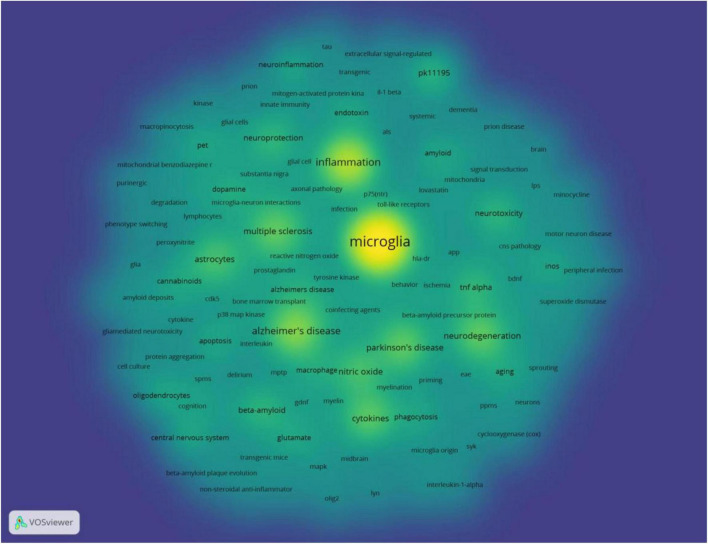
Co-occurrence analysis of frequently occurred author keywords, weighed by occurrence, density map.

The full counting co-occurrence analysis of author keywords showed that there were 143 keywords when ranked by occurrence, the top ten frequently occurred keywords were “microglia,” “inflammation,” “Alzheimer’s disease,” “cytokines,” “Parkinson’s disease,” “neurodegeneration,” “multiple sclerosis,” “nitric oxide,” “astrocytes,” and “neurotoxicity,” The timeline map showed that the most frequently used keywords “inflammation,” “Alzheimer’s disease,” “multiple sclerosis,” and “Parkinson’s disease” frequently occurred around 2005. The “cytokines,” “nitric oxide,” “astrocytes,” frequently occurred around 2000. While “dementia,” “delirium,” “priming,” “myelin,” “axonal pathology,” “phenotype switching” frequently occurred around 2010 (Figure 11). The frequently used keywords were shown in the density map (Figure 12).

**FIGURE 13:**
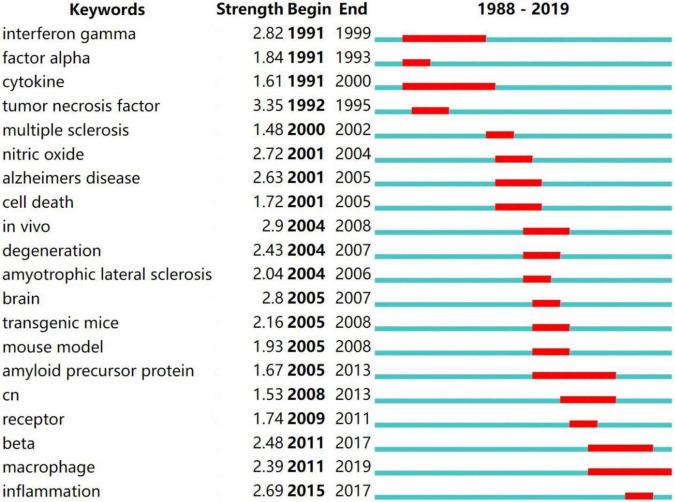
Burst detection of author keywords.

The burst detection of author keywords showed that “interferon-gamma” burst from 1991 to 1999, “Cytokine” burst from 1991 to 2000, “Alzheimer’s disease” and “cell death” burst from 2001 to 2005, “*in vivo*” burst from 2004 to 2008, “amyloid precursor protein” burst from 2005 to 2013, “beta” burst from 2011 to 2017, “macrophage” burst from 2011 to 2019, “inflammation” burst from 2015 to 2017 (Figure 13).

## Discussion

This bibliometric and visualized study identified and analyzed the top 100 highly cited articles focusing on the role of microglia and NDs, to our knowledge, we first analyzed the top-cited articles within the field using a bibliometric method and presented some interesting and useful conclusions. Revealing the highly cited authors, institutions, and countries help readers quickly locate the most productive and influential contributors. It must be emphasized that the citations can not fully represent the real academic values, “highly cited”were not equal to “influential”, the impact should be evaluated comprehensively. Even though the representative value of the extracted hotspots from 100 articles is limited, the results of this study can still answer the following questions: What are the highly cited articles mainly focused on? what may be the hotspots in this research field?

The limitations of the study design must be clarified. This is a citation-based bibliometric analysis, most of the results were conducted *via* visualized analysis, the standard of the evaluation was sole and the results may not be very persuasive. The academic value of an article should be comprehensively evaluated, except for the cited times, the contribution to the field, whether it added new to the existing knowledge, whether it can guide the clinical management of the diseases should be considered. Besides, the impact factors, h-index, reputations, and rate of self-citation of journals should be considered. We analyzed the author keywords by the co-occurrence analysis and burst of keywords, the detailed research topics and key points of these articles were not retracted. Due to the limited number of author keywords, the burst detection and timeline map of keywords may not represent the real hotspots. Furthermore, the total citations can be significantly influenced by the age of literature, which may lead to bias when screening highly cited articles. Despite these shortcomings, citation analysis is the most widely used and subjective method in bibliometric analysis.

The peak of publications of the highly cited articles was during the first decade of the 21st century, indicating that the period was the golden years of highly cited microglia research. The citation of 2021 was not shown on the map because it has not been fully counted and the results can not represent the real trend. It must be emphasized that the total cited times of articles that were published after the 2010s can be underestimated, to balance the citations and age of literature, we introduced citation density to evaluate the average citations yearly. The results showed that the citation densities raised yearly, articles with high citation density frequently occurred after 2010, articles with the highest citation densities frequently appeared in the recent 5 years, indicating that recently published articles were more prone to be cited. In terms of yearly total citations, the number of yearly citations quickly raised since 2015, suggesting that the number of original research greatly boomed in recent 7 years. The citation number of 2021 was not presented in the trend map because it has not been fully counted.

The United States had the largest record counts among the top 100 cited articles, which owns the overwhelming number of citations and record counts, indicating that the United States was the most productive and influential country in this field. With the aging of the global population, the burden of NDs is increasing yearly (Chee and Solito, 2021). In the United States, Alzheimer’s disease affected five million people and caused a huge amount of healthcare costs (Hurd et al., 2013), it has been predicted that the number of dementia in the United States will increase by 35% by 2030 (Petersen et al., 2018). Parkinson’s disease has been reported with the prevalence of 4% in men and 2% in women in the 85 years old North American population (Marras et al., 2018). Multiple sclerosis affects 2–3 million people globally (Browne et al., 2014) (Vaughn et al., 2019), especially minorities in the United States (Ventura et al., 2017; Wallin et al., 2019). The United States owns the largest number of famous universities and leads the tide of neurodegenerative research around the world. We found that nearly all of the top 10 highly cited institutions belong to the United States (9/10). More than half of the highly cited authors were from the United States (7/10), more than half of the most frequently cited authors in the references were from the United States (6/10). Harvard University was the institution with overwhelming cited times among all institutions, reflecting its authority in the field of microglia and NDs.

The top-cited articles were most often published in top journals within the research field, including Nature, Science, and Cell, as well as the authoritative journals in the field of neuroscience, including the Journal of Neuroscience, Nature Neuroscience, Nature Medicine, and Glia, suggesting that these journals mostly tend to publish original research in microglia and NDs. The distribution of highly cited journals in the references was similar to the published journals. It’s worth noting that the Journal of Neuroscience, though has a lower impact factor compared to these top journals, was both the largest publisher and the most frequently cited journals in the references, suggesting its authority in this field. The impact factor of one journal was calculated by citations and the number of published articles, however, it should not be considered as the most important standard when evaluating the academic value of journals. Revealing the highly cited journals help readers quickly locate the most often published and cited publishers in this field, which can be a reference when submitting their papers and retrieving the literature.

The hotspots were extracted by co-occurrence analysis and burst detection of author keywords. The Citespace software, developed by Doctor Chaomei Chen (Chen et al., 2012), was widely used in the bibliometric analysis, especially famous for its burst detection function. The results showed that the most interesting NDs were Alzheimer’s Disease, followed by Parkinson’s Disease, the third was Multiple Sclerosis, the trend was consistent with the morbidity and global epidemiology of NDs. Keywords that described the symptom of Alzheimer’s disease were most frequently used, suggesting that Alzheimer’s disease was the hotspot in the research of NDs. The microglia was the main inflammatory cell during the pathogenesis and development of NDs (Yeung et al., 2017). Not surprisingly, the original research most frequently focused on inflammation, and the keywords related to inflammations burst in recent years.

To emphasize the clinical guiding significance, original clinical research was also included for analysis. Besides, we extracted and listed the top 10 cited reviews in this field (not among the top 100 articles) and summarized their major topics (Supplementary Table 2). Among the top 10 most cited reviews, five of them were from the United States, they focused on the mechanism of neuroinflammation in Alzheimer’s disease (Akiyama et al., 2000), neurotoxicity mediated by microglia in NDs (Block et al., 2007), oxidative stress (Andersen, 2004), and molecular mechanism of blood-brain barrier damage (Zlokovic, 2008). In two reviews from England, the authors investigated the interaction between microglia and oligodendrocytes and the mechanism of microglia in neuroinflammatory demyelination in multiple sclerosis (Fawcett and Asher, 1999; Compston and Coles, 2008). Two reviews from Germany discussed the mechanism of neuroinflammation in Alzheimer’s disease and the specific pathway of glutathione, an important factor of oxidative stress in the nervous system (Dringen, 2000; Heneka et al., 2015). One review summarized the detailed process of neuroinflammatory response in Parkinson’s disease and preliminarily presented the research progress and future research direction of targeting microglial inflammatory factors to alleviate the clinical symptoms of Parkinson’s disease (Hirsch and Hunot, 2009). Though the highly cited reviews were not included for analysis in this study, they represent the currently most focused topics in the field of microglia and NDs. Regarding the epidemiology of the NDs, the incidence of Alzheimer’s disease, dementia, and cognitive impairment is increasing yearly. Previous studies have confirmed the toxic effect of microglial inflammation on neurons. Therefore, it is a general trend to clarify the role of microglia in dementia.

Regarding the mechanisms, activated microglia continuously secrete inflammatory factors and reactive oxygen species to attack neurons, resulting in neuronal damage (Fiebich et al., 2014; Španić et al., 2019). Microglia may also respond to demyelination of oligodendrocyte precursor cells by phagocytosis of myelin fragments, remodeling extracellular matrix and secreting nutritional factors needed by oligodendrocytes, and promoting the process of neuronal extubation and remyelination (Tay et al., 2017). In addition, microglia have a Toll-like Receptor on the surface, which can effectively mediate antigen recognition (Schetters et al., 2017). Activated microglia also have a lysosomal mechanism, which helps antigens recognize and express MHC-class II costimulatory molecules (Verkhratsky et al., 2021) needed to present myelin antigen peptides to central nervous system antigen-specific CD4^+^ T cells. The mechanisms were not the main purpose of this bibliometric analysis, but it still helps readers better understand the current research trends.

Inflammatory demyelination and neuronal degeneration are the main pathological changes of multiple sclerosis. Microglia is crucial in this pathological change, neuroinflammation is the hot area in the current research, which is consistent with the major conclusion of this study. In short, microglia mainly participate in the process of oxidative stress and antigen presentation, which affect the permeability and integrity of the blood–brain barrier, and eventually lead to the disorder of the immune internal environment of the central nervous system. Therefore, microglia may have different phenotypes, functions, and states under different pathological stimuli, but whether their effects are pathogenic or protective, or both, is controversial (Prinz et al., 2011).

The development of single-cell sequencing and genomics techniques has further enabled the classification and identification of microglia from phagocytes of other sources, which is conducive to the development of targeted drugs for microglia (Miron et al., 2013). Some experimental studies revealed that macrophages have a certain effect on myelin regeneration, but the specific process is not clear and needs further research (Ruckh et al., 2012; Sominsky et al., 2018). For clinicians, gathering top-cited articles may contribute to the clinical transformation of the basic research of diagnosis, clinical decision-making, as well as explore the diagnostic markers of clinical diseases and drugs targeting specific phenotypic functions of microglia.

The bibliometric analysis and visualized study have some limitations. First, the retrieve of literature was based on the WOS website only, the representative value of extracted results was limited. Second, this is a citation-based bibliometric analysis, the screening of articles, rank of authors, institutions, journals, and countries was mostly based on total cited times, the “highly cited” are not equal to “influential,” thus the conclusion can be not very persuasive. Furthermore, the citations can be significantly influenced by age of literature, articles published recently may be missed though they have high academic value. Third, the hotspots that were extracted from keywords may not represent the real research interests because the number of total keywords was small. Fourth, the bibliometric analysis mainly focused on quantitatively analyzing the characteristics of the literature, the detailed context of these articles were not fully investigated and summarized, as a result, the conclusions were superficial. Fifth, the majority of articles included in this study were experimental research, we aimed to reveal the trend in the original research, though clinical articles were included and top-cited reviews were discussed, limited useful information can be gathered by clinicians.

## Conclusion

This bibliometric and visualized analysis investigated the 100 top-cited original studies concentrated on the role of microglia in NDs. Revealing the highly cited articles help readers quickly locate the most productive and influential contributors, as well as gather the hotspots and latest trend in this field. The United States was the biggest contributor, Harford University was the most influential institution. Journal of Neuroscience was the most often published and cited journal. Alzheimer’s disease was the hotspot of microglia-related study. The recent research mainly focused on inflammation.

## Data Availability Statement

The original contributions presented in the study are included in the article/Supplementary Material, further inquiries can be directed to the corresponding author.

## Author Contributions

MS and XL: conceptualization and writing of the original draft. JS: data curation. HW: software. QX: conceptualization and writing—review and editing; MW: conceptualization and project administration. All authors contributed to the article and approved the submitted version.

## Conflict of Interest

The authors declare that the research was conducted in the absence of any commercial or financial relationships that could be construed as a potential conflict of interest.

## Publisher’s Note

All claims expressed in this article are solely those of the authors and do not necessarily represent those of their affiliated organizations, or those of the publisher, the editors and the reviewers. Any product that may be evaluated in this article, or claim that may be made by its manufacturer, is not guaranteed or endorsed by the publisher.
