# Supplementary material for: The Top-Cited Original Articles on the Role of Microglia in Neurodegenerative Diseases: A Bibliometric and Visualized Study
Source: Front Aging Neurosci. 2022 Apr 5;14:869964. doi: 10.3389/fnagi.2022.869964 (PMC9037152; doi:10.3389/fnagi.2022.869964)
Supplement: Supplementary file 1 [file Table_1.DOCX]

**Supplementary Table 1** List of the top 100 most-cited original articles on the role of microglia in neurodegenerative disease

| Number | Article | Year | Citations |
| --- | --- | --- | --- |
| 1 | Ginhoux F, Greter M, Leboeuf M, Nandi S, See P, Gokhan S, Mehler MF, Conway SJ, Ng LG, Stanley ER, Samokhvalov IM, Merad M. Fate Mapping Analysis Reveals That Adult Microglia Derive from Primitive Macrophages. Science 330(6005): 841-845. | 2010 | 2699 |
| 2 | Liddelow SA, Guttenplan KA, Larke LEC, Bennett FC, Bohlen CJ, Schirmer L, Bennett ML, Munch AE, Chung WS, Peterson TC, Wilton DK, Frouin A, Napier BA, Panicker N, Kumar M, Buckwalter MS, Rowitch DH, Dawson VL, Dawson TM, Stevens B, Barres B. Neurotoxic reactive astrocytes are induced by activated microglia. Nature 541(7638): 481-487. | 2017 | 2516 |
| 3 | Mcgeer PL, Itagaki S, Boyes BE, Mcgeer EG. Reactive Microglia Are Positive for Hla-dr in the Substantia Nigra of Parkinsons and Alzheimers-disease Brains. Neurology 38(8): 1285-1291. | 1988 | 2058 |
| 4 | Yan SD, Chen X, Fu J, Chen M, Zhu HJ, Roher A, Slattery T, Zhao L, Nagashima M, Morser J, Migheli A, Nawroth P, Stern D, Schmidt AM. RAGE and amyloid-beta peptide neurotoxicity in Alzheimer's disease. Nature 382(6593): 685-691. | 1996 | 1720 |
| 5 | Bard F, Cannon C, Barbour R, Burke RL, Games D, Grajeda H, Guido T, Hu K, Huang JP, Johnson-Wood K, Khan K, Kholodenko D, Lee M, Lieberburg I, Motter R, Nguyen M, Soriano F, Vasquez N, Weiss K, Welch B, Seubert P, Schenk D, Yednock T. Peripherally administered antibodies against amyloid beta-peptide enter the central nervous system and reduce pathology in a mouse model of Alzheimer disease. Nature Medicine 6(8): 916-919. | 2000 | 1690 |
| 6 | Halle A, Hornung V, Petzold GC, Stewart CR, Monks BG, Reinheckel T, Fitzgerald KA, Latz E, Moore KJ, Golenbock DT. The NALP3 inflammasome is involved in the innate immune response to amyloid-beta. Nature Immunology 9(8): 857-865. | 2008 | 1613 |
| 7 | Keren-Shaul H, Spinrad A, Weiner A, Matcovitch-Natan O, Dvir-Szternfeld R, Ulland TK, David E, Baruch K, Lara-Astaiso D, Toth B, Itzkovitz S, Colonna M, Schwartz M, Amit I. A Unique Microglia Type Associated with Restricting Development of Alzheimer's Disease. Cell 169(7): 1276. | 2017 | 1459 |
| 8 | Heneka MT, Kummer MP, Stutz A, Delekate A, Schwartz S, Vieira-Saecker A, Griep A, Axt D, Remus A, Tzeng TC, Gelpi E, Halle A, Korte M, Latz E, Golenbock DT. NLRP3 is activated in Alzheimer's disease and contributes to pathology in APP/PS1 mice. Nature 493(7434): 674-+. | 2013 | 1399 |
| 9 | Qin LY, Wu XF, Block ML, Liu YX, Breese GR, Hong JS, Knapp DJ, Crews FT. Systemic LPS causes chronic neuroinflammation and progressive neurodegeneration. Glia 55(5): 453-462. | 2007 | 1396 |
| 10 | Sampson TR, Debelius JW, Thron T, Janssen S, Shastri GG, Ilhan ZE, Challis C, Schretter CE, Rocha S, Gradinaru V, Chesselet MF, Keshavarzian A, Shannon KM, Krajmalnik-Brown R, Wittung-Stafshede P, Knight R, Mazmanian SK. Gut Microbiota Regulate Motor Deficits and Neuroinflammation in a Model of Parkinson's Disease. Cell 167(6): 1469-+. | 2016 | 1383 |
| 11 | Hong S, Beja-Glasser VF, Nfonoyim BM, Frouin A, Li SM, Ramakrishnan S, Merry KM, Shi QQ, Rosenthal A, Barres BA, Lemere CA, Selkoe DJ, Stevens B. Complement and microglia mediate early synapse loss in Alzheimer mouse models. Science 352(6286): 712-716. | 2016 | 1222 |
| 12 | Kutzelnigg A, Lucchinetti CF, Stadelmann C, Bruck W, Rauschka H, Bergmann M, Schmidbauer M, Parisi JE, Lassmann H. Cortical demyelination and diffuse white matter injury in multiple sclerosis. Brain 128(2705-2712. | 2005 | 1203 |
| 13 | Meda L, Cassatella Ma, Szendrei Gi, Otvos L, Baron P, Villalba M, Ferrari D, Rossi F. Activation of Microglial Cells by Beta-amyloid Protein and Interferon-gamma. Nature 374(6523): 647-650. | 1995 | 1198 |
| 14 | Boillee S, Yamanaka K, Lobsiger CS, Copeland NG, Jenkins NA, Kassiotis G, Kollias G, Cleveland DW. Onset and progression in inherited ALS determined by motor neurons and microglia. Science 312(5778): 1389-1392. | 2006 | 1179 |
| 15 | Yoshiyama Y, Higuchi M, Zhang B, Huang SM, Iwata N, Saido TC, Maeda J, Suhara T, Trojanowski JQ, Lee VMY. Synapse loss and microglial activation precede tangles in a P301S tauopathy mouse model. Neuron 53(3): 337-351. | 2007 | 1131 |
| 16 | Cardona AE, Pioro EP, Sasse ME, Kostenko V, Cardona SM, Dijkstra IM, Huang DR, Kidd G, Dombrowski S, Dutta R, Lee JC, Cook DN, Jung S, Lira SA, Littman DR, Ransohoff RM. Control of microglial neurotoxicity by the fractalkine receptor. Nature Neuroscience 9(7): 917-924. | 2006 | 1048 |
| 17 | Peterson JW, Bo L, Mork S, Chang A, Trapp BD. Transected neurites, apoptotic neurons, and reduced inflammation in cortical multiple sclerosis lesions. Annals of Neurology 50(3): 389-400. | 2001 | 1046 |
| 18 | Chao Cc, Hu Sx, Molitor Tw, Shaskan Eg, Peterson Pk. Activated Microglia Mediate Neuronal Cell Injury Via a Nitric-oxide Mechanism. Journal of Immunology 149(8): 2736-2741. | 1992 | 1021 |
| 19 | Ajami B, Bennett JL, Krieger C, Tetzlaff W, Rossi FMV. Local self-renewal can sustain CNS microglia maintenance and function throughout adult life. Nature Neuroscience 10(12): 1538-1543. | 2007 | 1020 |
| 20 | Wu DC, Jackson-Lewis V, Vila M, Tieu K, Teismann P, Vadseth C, Choi DK, Ischiropoulos H, Przedborski S. Blockade of microglial activation is neuroprotective in the 1-methyl-4-phenyl-1,2,3,6-tetrahydropyridine mouse model of Parkinson disease. Journal of Neuroscience 22(5): 1763-1771. | 2002 | 1013 |
| 21 | Miron VE, Boyd A, Zhao JW, Yuen TJ, Ruckh JM, Shadrach JL, Van Wijngaarden P, Wagers AJ, Williams A, Franklin RJM, Ffrench-Constant C. M2 microglia and macrophages drive oligodendrocyte differentiation during CNS remyelination. Nature Neuroscience 16(9): 1211-U1275. | 2013 | 961 |
| 22 | Simard AR, Soulet D, Gowing G, Julien JP, Rivest S. Bone marrow-derived microglia play a critical role in restricting senile plaque formation in Alzheimer's disease. Neuron 49(4): 489-502. | 2006 | 944 |
| 23 | Youssef S, Stuve O, Patarroyo JC, Ruiz PJ, Radosevich JL, Hur EM, Bravo M, Mitchell DJ, Sobel RA, Steinman L, Zamvil SS. The HMG-CoA reductase inhibitor, atorvastatin, promotes a Th2 bias and reverses paralysis in central nervous system autoimmune disease. Nature 420(6911): 78-84. | 2002 | 929 |
| 24 | Liu B, Hong JS. Role of microglia in inflammation-mediated neurodegenerative diseases: Mechanisms and strategies for therapeutic intervention. Journal of Pharmacology and Experimental Therapeutics 304(1): 1-7. | 2003 | 924 |
| 25 | Boje Km, Arora Pk. Microglial-produced Nitric-oxide and Reactive Nitrogen-oxides Mediate Neuronal Cell-death. Brain Research 587(2): 250-256. | 1992 | 849 |
| 26 | Krasemann S, Madore C, Cialic R, Baufeld C, Calcagno N, El Fatimy R, Beckers L, O'loughlin E, Xu Y, Fanek Z, Greco DJ, Smith ST, Tweet G, Humulock Z, Zrzavy T, Conde-Sanroman P, Gacias M, Weng ZP, Chen H, Tjon E, Mazaheri F, Hartmann K, Madi A, Ulrich JD, Glatzel M, Worthmann A, Heeren J, Budnik B, Lemere C, Ikezu T, Heppner FL, Litvak V, Holtzman DM, Lassmann H, Weiner HL, Ochando J, Haass C, Butovsky O. The TREM2-APOE Pathway Drives the Transcriptional Phenotype of Dysfunctional Microglia in Neurodegenerative Diseases. Immunity 47(3): 566-+. | 2017 | 843 |
| 27 | Zhang W, Wang TG, Pei Z, Miller DS, Wu XF, Block ML, Wilson B, Zhang WQ, Zhou Y, Hong JS, Zhang J. Aggregated alpha-synuclein activates microglia: a process leading to disease progression in Parkinson's disease. Faseb Journal 19(6): 533-542. | 2005 | 832 |
| 28 | Hickman SE, Kingery ND, Ohsumi TK, Borowsky ML, Wang LC, Means TK, El Khoury J. The microglial sensome revealed by direct RNA sequencing. Nature Neuroscience 16(12): 1896-1905. | 2008 | 830 |
| 29 | Bezzi P, Domercq M, Brambilla L, Galli R, Schols D, De Clercq E, Vescovi A, Bagetta G, Kollias G, Meldolesi J, Volterra A. CXCR4-activated astrocyte glutamate release via TNFa: amplification by microglia triggers neurotoxicity. Nature Neuroscience 4(7): 702-710. | 2001 | 823 |
| 30 | Hickman SE, Allison EK, El Khoury J. Microglial dysfunction and defective beta-amyloid clearance pathways in aging Alzheimer's disease mice. Journal of Neuroscience 28(33): 8354-8360. | 2013 | 822 |
| 31 | Cagnin A, Brooks DJ, Kennedy AM, Gunn RN, Myers R, Turkheimer FE, Jones T, Banati RB. In-vivo measurement of activated microglia in dementia. Lancet 358(9280): 461-467. | 2001 | 801 |
| 32 | Tikka T, Fiebich BL, Goldsteins G, Keinanen R, Koistinaho J. Minocycline, a tetracycline derivative, is neuroprotective against excitotoxicity by inhibiting activation and proliferation of microglia. Journal of Neuroscience 21(8): 2580-2588. | 2001 | 787 |
| 33 | Dickson Dw, Lee Sc, Mattiace La, Yen Shc, Brosnan C. Microglia and Cytokines in Neurological Disease, with Special Reference to Aids and Alzheimers-disease. Glia 7(1): 75-83. | 1993 | 784 |
| 34 | Barnett MH, Prineas JW. Relapsing and remitting multiple sclerosis: Pathology of the newly forming lesion. Annals of Neurology 55(4): 458-468. | 2004 | 769 |
| 35 | Wang YM, Cella M, Mallinson K, Ulrich JD, Young KL, Robinette ML, Gilfillan S, Krishnan GM, Sudhakar S, Zinselmeyer BH, Holtzman DM, Cirrito JR, Colonna M. TREM2 Lipid Sensing Sustains the Microglial Response in an Alzheimer's Disease Model. Cell 160(6): 1061-1071. | 2015 | 753 |
| 36 | Meyer-Luehmann M, Spires-Jones TL, Prada C, Garcia-Alloza M, De Calignon A, Rozkalne A, Koenigsknecht-Talboo J, Holtzman DM, Bacskai BJ, Hyman BT. Rapid appearance and local toxicity of amyloid-beta plaques in a mouse model of Alzheimer's disease. Nature 451(7179): 720-U725. | 2008 | 751 |
| 37 | Bsibsi M, Ravid R, Gveric D, Van Noort JM. Broad expression of Toll-like receptors in the human central nervous system. Journal of Neuropathology and Experimental Neurology 61(11): 1013-1021. | 2002 | 750 |
| 38 | Itagaki S, Mcgeer Pl, Akiyama H, Zhu S, Selkoe D. Relationship of Microglia and Astrocytes to Amyloid Deposits of Alzheimer-disease. Journal of Neuroimmunology 24(3): 173-182. | 1989 | 736 |
| 39 | Gerhard A, Pavese N, Hotton G, Turkheimer F, Es M, Hammers A, Eggert K, Oertel W, Banati RB, Brooks DJ. In vivo imaging of microglial activation with C-11 (R)-PK11195 PET in idiopathic Parkinson's disease. Neurobiology of Disease 21(2): 404-412. | 2006 | 730 |
| 40 | Asai H, Ikezu S, Tsunoda S, Medalla M, Luebke J, Haydar T, Wolozin B, Butovsky O, Kugler S, Ikezu T. Depletion of microglia and inhibition of exosome synthesis halt tau propagation. Nature Neuroscience 18(11): 1584-1593. | 2015 | 729 |
| 41 | Streit WJ. Microglia as neuroprotective, immunocompetent cells of the CNS. Glia 40(2): 133-139. | 2002 | 725 |
| 42 | Emmanouilidou E, Melachroinou K, Roumeliotis T, Garbis SD, Ntzouni M, Margaritis LH, Stefanis L, Vekrellis K. Cell-Produced alpha-Synuclein Is Secreted in a Calcium-Dependent Manner by Exosomes and Impacts Neuronal Survival. Journal of Neuroscience 30(20): 6838-6851. | 2010 | 718 |
| 43 | Merrill Je, Ignarro Lj, Sherman Mp, Melinek J, Lane Te. Microglial Cell Cytotoxicity of Oligodendrocytes is Mediated Through Nitric-oxide. Journal of Immunology 151(4): 2132-2141. | 1993 | 706 |
| 44 | Selmaj K, Raine Cs, Cannella B, Brosnan Cf. Identification of Lymphotoxin and Tumor-necrosis-factor in Multiple-sclerosis Lesions. Journal of Clinical Investigation 87(3): 949-954. | 1991 | 704 |
| 45 | Ajami B, Bennett JL, Krieger C, Mcnagny KM, Rossi FMV. Infiltrating monocytes trigger EAE progression, but do not contribute to the resident microglia pool. Nature Neuroscience 14(9): 1142-U1263. | 2011 | 701 |
| 46 | Kim WG, Mohney RP, Wilson B, Jeohn GH, Liu B, Hong JS. Regional difference in susceptibility to lipopolysaccharide-induced neurotoxicity in the rat brain: Role of microglia. Journal of Neuroscience 20(16): 6309-6316. | 2000 | 700 |
| 47 | Balashov KE, Rottman JB, Weiner HL, Hancock WW. CCR5(+) and CXCR3(+) T cells are increased in multiple sclerosis and their ligands MIP-1 alpha and IP-10 are expressed in demyelinating brain lesions. Proceedings of the National Academy of Sciences of the United States of America 96(12): 6873-6878. | 1999 | 700 |
| 48 | Long TC, Saleh N, Tilton RD, Lowry GV, Veronesi B. Titanium dioxide (P25) produces reactive oxygen species in immortalized brain microglia (BV2): Implications for nanoparticle neurotoxicity. Environmental Science & Technology 40(14): 4346-4352. | 2006 | 685 |
| 49 | Qiu WQ, Walsh DM, Ye Z, Vekrellis K, Zhang JM, Podlisny MB, Rosner MR, Safavi A, Hersh LB, Selkoe DJ. Insulin-degrading enzyme regulates extracellular levels of amyloid beta-protein by degradation. Journal of Biological Chemistry 273(49): 32730-32738. | 1998 | 684 |
| 50 | Wyss-Coray T, Loike JD, Brionne TC, Lu E, Anankov R, Yan FR, Silverstein SC, Husemann J. Adult mouse astrocytes degrade amyloid-beta in vitro and in situ. Nature Medicine 9(4): 453-457. | 2003 | 674 |
| 51 | Bhat NR, Zhang PS, Lee JC, Hogan EL. Extracellular signal-regulated kinase and p38 subgroups of mitogen-activated protein kinases regulate inducible nitric oxide synthase and tumor necrosis factor-alpha gene expression in endotoxin-stimulated primary glial cultures. Journal of Neuroscience 18(5): 1633-1641. | 1998 | 672 |
| 52 | Holmes C, Cunningham C, Zotova E, Woolford J, Dean C, Kerr S, Culliford D, Perry VH. Systemic inflammation and disease progression in Alzheimer disease. Neurology 73(10): 768-774. | 2009 | 668 |
| 53 | Jiang Q, Lee CYD, Mandrekar S, Wilkinson B, Cramer P, Zelcer N, Mann K, Lamb B, Willson TM, Collins JL, Richardson JC, Smith JD, Comery TA, Riddell D, Holtzman DM, Tontonoz P, Landreth GE. ApoE promotes the proteolytic degradation of A beta. Neuron 58(5): 681-693. | 2008 | 655 |
| 54 | Cannella B, Raine Cs. the Adhesion Molecule and Cytokine Profile of Multiple-sclerosis Lesions. Annals of Neurology 37(4): 424-435. | 1995 | 650 |
| 55 | Saijo K, Winner B, Carson CT, Collier JG, Boyer L, Rosenfeld MG, Gage FH, Glass CK. A Nurr1/CoREST Pathway in Microglia and Astrocytes Protects Dopaminergic Neurons from Inflammation-Induced Death. Cell 137(1): 47-59. | 2009 | 637 |
| 56 | Elkhoury J, Hickman SE, Thomas CA, Cao L, Silverstein SC, Loike JD. Scavenger receptor-mediated adhesion of microglia to beta-amyloid fibrils. Nature 382(6593): 716-719. | 1996 | 627 |
| 57 | Chen J, Zhou YG, Mueller-Steiner S, Chen LF, Kwon H, Yi SL, Mucke L, Li G. SIRT1 protects against microglia-dependent amyloid-beta toxicity through inhibiting NF-kappa B signaling. Journal of Biological Chemistry 280(48): 40364-40374. | 2005 | 619 |
| 58 | Ouchi Y, Yoshikawa E, Sekine Y, Futatsubashi M, Kanno T, Ogusu T, Torizuka T. Microglial activation and dopamine terminal loss in early Parkinson's disease. Annals of Neurology 57(2): 168-175. | 2005 | 572 |
| 59 | Gao HM, Jiang J, Wilson B, Zhang W, Hong JS, Liu B. Microglial activation-mediated delayed and progressive degeneration of rat nigral dopaminergic neurons: relevance to Parkinson's disease. Journal of Neurochemistry 81(6): 1285-1297. | 2002 | 572 |
| 60 | Ling Ea, Wong Wc. the Origin and Nature of Ramified and Ameboid Microglia - a Historical Review and Current Concepts. Glia 7(1): 9-18. | 1993 | 570 |
| 61 | Johnson VE, Stewart JE, Begbie FD, Trojanowski JQ, Smith DH, Stewart W. Inflammation and white matter degeneration persist for years after a single traumatic brain injury. Brain 136(28-42. | 2013 | 569 |
| 62 | Combs CK, Karlo JC, Kao SC, Landreth GE. beta-Amyloid stimulation of microglia and monocytes results in TNF alpha-dependent expression of inducible nitric oxide synthase and neuronal apoptosis. Journal of Neuroscience 21(4): 1179-1188. | 2001 | 568 |
| 63 | Heppner FL, Greter M, Marino D, Falsig J, Raivich G, Hovelmeyer N, Waisman A, Rulicke T, Prinz M, Priller J, Becher B, Aguzzi A. Experimental autoimmune encephalomyelitis repressed by microglial paralysis. Nature Medicine 11(2): 146-152. | 2005 | 563 |
| 64 | Godbout JP, Chen J, Abraham J, Richwine AF, Berg BM, Kelley KW, Johnson RW. Exaggerated neuroinflammation and sickness behavior in aged mice after activation of the peripheral innate immune system. Faseb Journal 19(7): 1329-+. | 2005 | 560 |
| 65 | Kuhlmann T, Miron V, Cuo Q, Wegner C, Antel J, Bruck W. Differentiation block of oligodendroglial progenitor cells as a cause for remyelination failure in chronic multiple sclerosis. Brain 131(1749-1758. | 2008 | 557 |
| 66 | Ramirez BG, Blazquez C, Del Pulgar TG, Guzman N, De Ceballos MaL. Prevention of Alzheimer's disease pathology by cannabinoids: Neuroprotection mediated by blockade of microglial activation. Journal of Neuroscience 25(8): 1904-1913. | 2005 | 554 |
| 67 | Jacobsen JS, Wu CC, Redwine JM, Comery TA, Arias R, Bowlby M, Martone R, Morrison JH, Pangalos MN, Reinhart PH, Bloom FE. Early-onset behavioral and synaptic deficits in a mouse model of Alzheimer's disease. Proceedings of the National Academy of Sciences of the United States of America 103(13): 5161-5166. | 2006 | 552 |
| 68 | Minghetti L. Cyclooxygenase-2 (COX-2) in inflammatory and degenerative brain diseases. Journal of Neuropathology and Experimental Neurology 63(9): 901-910. | 2004 | 547 |
| 69 | Paresce DM, Ghosh RN, Maxfield FR. Microglial cells internalize aggregates of the Alzheimer's disease amyloid beta-protein via a scavenger receptor. Neuron 17(3): 553-565. | 1996 | 547 |
| 70 | Bal-Price A, Brown GC. Inflammatory neurodegeneration mediated by nitric oxide from activated glia-inhibiting neuronal respiration, causing glutamate release and excitotoxicity. Journal of Neuroscience 21(17): 6480-6491. | 2001 | 545 |
| 71 | Jack CS, Arbour N, Manusow J, Montgrain V, Blain M, Mccrea E, Shapiro A, Antel JP. TLR signaling tailors innate immune responses in human microglia and astrocytes. Journal of Immunology 175(7): 4320-4330. | 2005 | 536 |
| 72 | Chang A, Nishiyama A, Peterson J, Prineas J, Trapp BD. NG2-positive oligodendrocyte progenitor cells in adult human brain and multiple sclerosis lesions. Journal of Neuroscience 20(17): 6404-6412. | 2000 | 534 |
| 73 | Lee HJ, Suk JE, Patrick C, Bae EJ, Cho JH, Rho S, Hwang D, Masliah E, Lee SJ. Direct Transfer of alpha-Synuclein from Neuron to Astroglia Causes Inflammatory Responses in Synucleinopathies. Journal of Biological Chemistry 285(12): 9262-9272. | 2010 | 528 |
| 74 | Anderson AC, Anderson DE, Bregoli L, Hastings WD, Kassam N, Lei C, Chandwaskar R, Karman J, Su EW, Hirashima M, Bruce JN, Kane LP, Kuchroo VK, Hafler DA. Promotion of tissue inflammation by the immune receptor Tim-3 expressed on innate immune cells. Science 318(5853): 1141-1143. | 2007 | 528 |
| 75 | Banati RB, Newcombe J, Gunn RN, Cagnin A, Turkheimer F, Heppner F, Price G, Wegner F, Giovannoni G, Miller DH, Perkin GD, Smith T, Hewson AK, Bydder G, Kreutzberg GW, Jones T, Cuzner ML, Myers R. The peripheral benzodiazepine binding site in the brain in multiple sclerosis - Quantitative in vivo imaging of microglia as a measure of disease activity. Brain 123(2321-2337. | 2000 | 523 |
| 76 | Lehnardt S, Lachance C, Patrizi S, Lefebvre S, Follett PL, Jensen FE, Rosenberg PA, Volpe JJ, Vartanian T. The toll-like receptor TLR4 is necessary for lipopolysaccharide-induced oligodendrocyte injury in the CNS. Journal of Neuroscience 22(7): 2478-2486. | 2002 | 522 |
| 77 | Barger SW, Harmon AD. Microglial activation by Alzheimer amyloid precursor protein and modulation by apolipoprotein E. Nature 388(6645): 878-881. | 1997 | 522 |
| 78 | Beers DR, Henkel JS, Xiao Q, Zhao WH, Wang JH, Yen AA, Siklos L, Mckercher SR, Appel SH. Wild-type microglia extend survival in PU.1 knockout mice with familial amyotrophic lateral sclerosis. Proceedings of the National Academy of Sciences of the United States of America 103(43): 16021-16026. | 2006 | 521 |
| 79 | Cunningham C, Wilcockson DC, Campion S, Lunnon K, Perry VH. Central and systemic endotoxin challenges exacerbate the local inflammatory response and increase neuronal death during chronic neurodegeneration. Journal of Neuroscience 25(40): 9275-9284. | 2005 | 521 |
| 80 | Yamasaki R, Lu HY, Butovsky O, Ohno N, Rietsch AM, Cialic R, Wu PM, Doykan CE, Lin J, Cotleur AC, Kidd G, Zorlu MM, Sun N, Hu WW, Liu LP, Lee JC, Taylor SE, Uehlein L, Dixon D, Gu JY, Floruta CM, Zhu M, Charo IF, Weiner HL, Ransohoff RM. Differential roles of microglia and monocytes in the inflamed central nervous system. Journal of Experimental Medicine 211(8): 1533-1549. | 2014 | 520 |
| 81 | Takeuchi H, Jin SJ, Wang JY, Zhang GQ, Kawanokuchi J, Kuno R, Sonobe Y, Mizuno T, Suzumura A. Tumor necrosis factor-alpha induces neurotoxicity via glutamate release from hemichannels of activated microglia in an autocrine manner. Journal of Biological Chemistry 281(30): 21362-21368. | 2006 | 520 |
| 82 | Turner MR, Cagnin A, Turkheimer FE, Miller CCJ, Shaw CE. Evidence of widespread cerebral microglial activation in amyotrophic lateral sclerosis: an C-11 (R)-PK11195 positron emission tomography study. Neurobiology of Disease 15(3): 601-609. | 2004 | 517 |
| 83 | Mcmahon EJ, Bailey SL, Castenada CV, Waldner H, Miller SD. Epitope spreading initiates in the CNS in two mouse models of multiple sclerosis. Nature Medicine 11(3): 335-339. | 2005 | 513 |
| 84 | Hammond TR, Dufort C, Dissing-Olesen L, Giera S, Young A, Wysoker A, Walker AJ, Gergits F, Segel M, Nemesh J, Marsh SE, Saunders A, Macosko E, Ginhoux F, Chen JM, Franklin RJM, Piao XH, Mccarroll SA, Stevens B. Single-Cell RNA Sequencing of Microglia throughout the Mouse Lifespan and in the Injured Brain Reveals Complex Cell-State Changes. Immunity 50(1): 253-+. | 2019 | 508 |
| 85 | Wu DC, Teismann P, Tieu K, Vila M, Jackson-Lewis V, Ischiropoulos H, Przedborski S. NADPH oxidase mediates oxidative stress in the 1-methyl-4-phenyl-1,2,3,6-tetrahydropyridine model of Parkinson's disease. Proceedings of the National Academy of Sciences of the United States of America 100(10): 6145-6150. | 2003 | 501 |
| 86 | Pahan K, Sheikh FG, Namboodiri AMS, Singh I. Lovastatin and phenylacetate inhibit the induction of nitric oxide synthase and cytokines in rat primary astrocytes, microglia, and macrophages. Journal of Clinical Investigation 100(11): 2671-2679. | 1997 | 501 |
| 87 | Batchelor PE, Liberatore GT, Wong JYF, Porritt MJ, Frerichs F, Donnan GA, Howells DW. Activated macrophages and microglia induce dopaminergic sprouting in the injured striatum sand express brain-derived neurotrophic factor and glial cell line-derived neurotrophic factor. Journal of Neuroscience 19(5): 1708-1716. | 1999 | 499 |
| 88 | Kitazawa M, Oddo S, Yamasaki TR, Green KN, Laferla FM. Lipopolysaccharide-induced inflammation exacerbates tau pathology by a cyclin-dependent kinase 5-mediated pathway in a transgenic model of Alzheimer's disease. Journal of Neuroscience 25(39): 8843-8853. | 2005 | 496 |
| 89 | Walter L, Franklin A, Witting A, Wade C, Xie YH, Kunos G, Mackie K, Stella N. Nonpsychotropic cannabinoid receptors regulate microglial cell migration. Journal of Neuroscience 23(4): 1398-1405. | 2003 | 496 |
| 90 | Fitzner D. Selective transfer of exosomes from oligodendrocytes to microglia by macropinocytosis. Journal of Cell Science. (2011) 124: 447-458. | 2011 | 495 |
| 91 | Frautschy SA, Yang FS, Irrizarry M, Hyman B, Saido TC, Hsiao K, Cole GM. Microglial response to amyloid plaques in APPsw transgenic mice. American Journal of Pathology 152(1): 307-317. | 1998 | 484 |
| 92 | Wyss-Coray T, Lin C, Yan FR, Yu GQ, Rohde M, Mcconlogue L, Masliah E, Mucke L. TGF-beta 1 promotes microglial amyloid-beta clearance and reduces plaque burden in transgenic mice. Nature Medicine 7(5): 612-618. | 2001 | 483 |
| 93 | Qin LY, Liu YX, Wang TG, Wei SJ, Block ML, Wilson B, Liu B, Hong JS. NADPH oxidase mediates lipopolysaccharide-induced neurotoxicity and proinflammatory gene expression in activated microglia. Journal of Biological Chemistry 279(2): 1415-1421. | 2004 | 482 |
| 94 | Geula C, Wu CK, Saroff D, Lorenzo A, Yuan ML, Yankner BA. Aging renders the brain vulnerable to amyloid beta-protein neurotoxicity. Nature Medicine 4(7): 827-831. | 1998 | 480 |
| 95 | Griffin Wst, Sheng Jg, Roberts Gw, Mrak Re. Interleukin-1 Expression in Different Plaque Types in Alzheimers-disease - Significance in Plaque Evolution. Journal of Neuropathology and Experimental Neurology 54(2): 276-281. | 1995 | 478 |
| 96 | Cunningham C. Microglia and neurodegeneration: The role of systemic inflammation. Glia 61(1): 71-90. | 2013 | 477 |
| 97 | Gosselin D, Skola D, Coufal NG, Holtman IR, Schlachetzki JCM,Sajti E, Jaeger BN, O'connor C, Fitzpatrick C, Pasillas MP, Pena M, Adair A, Gonda DD, Levy ML, Ransohoff RM, Gage FH, Glass CK. An environment-dependent transcriptional network specifies human microglia identity. Science 356(6344): 11. | 2017 | 468 |
| 98 | Ulland TK, Song WM, Huang SCC, Ulrich JD, Sergushichev A, Beatty WL, Loboda AA, Zhou YY, Caims NJ, Kambal A, Loginicheva E, Gilfillan S, Cella M, Virgin HW, Unanue ER, Wang YM, Artyomov MN, Holtzman DM, Colonna M. TREM2 Maintains Microglial Metabolic Fitness in Alzheimer's Disease. Cell 170(4): 649-+. | 2017 | 393 |
| 99 | Rothhammer V, Borucki DM, Tjon EC, Takenaka MC, Chao CC, Ardura-Fabregat A, De Lima KA, Gutierrez-Vazquez C, Hewson P, Staszewski O, Blain M, Healy L, Neziraj T, Borio M, Wheeler M, Dragin LL, Laplaud DA, Antel J, Alvarez JI, Prinz M, Quintana FJ. Microglial control of astrocytes in response to microbial metabolites. Nature 557(7707): 724-+. | 2018 | 363 |
| 100 | Abud EM, Ramirez RN, Martinez ES, Healy LM, Nguyen CHH, Newman SA, Yeromin AV, Scarfone VM, Marsh SE, Fimbres C, Caraway CA, Fote GM, Madany AM, Agrawal A, Kayed R, Gylys KH, Cahalan MD, Cummings BJ, Antel JP, Mortazavi A, Carson MJ, Poon WW, Blurton-Jones M. iPSC-Derived Human Microglia-like Cells to Study Neurological Diseases. Neuron 94(2): 278-+. | 2017 | 363 |

**Supplementary Table 2** List of the top 10 cited reviews on the role of microglia in neurodegenerative disease

| Number | Article | Year | Citations |
| --- | --- | --- | --- |
| 1 | Akiyama H, Barger S, Barnum S, Bradt B, Bauer J, Cole GM, Cooper NR, Eikelenboom P, Emmerling M, Fiebich BL, Finch CE, Frautschy S, Griffin WST, Hampel H, Hull M, Landreth G, Lue LF, Mrak R, Mackenzie IR, Mcgeer PL, O'banion MK, Pachter J, Pasinetti G, Plata-Salaman C, Rogers J, Rydel R, Shen Y, Streit W, Strohmeyer R, Tooyoma I, Van Muiswinkel FL, Veerhuis R, Walker D, Webster S, Wegrzyniak B, Wenk G, Wyss-Coray T, Neuroinflammation Working G. Inflammation and Alzheimer's disease. Neurobiology of Aging 21(3): 383-421. | 2000 | 3421 |
| 2 | Compston A, Coles A. Multiple sclerosis. Lancet 372(9648): 1502-1517. | 2008 | 3179 |
| 3 | Block ML, Zecca L, Hong JS. Microglia-mediated neurotoxicity: uncovering the molecular mechanisms. Nature Reviews Neuroscience 8(1): 57-69. | 2007 | 2808 |
| 4 | Heneka MT, Carson MJ, El Khoury J, Landreth GE, Brosseron F, Feinstein DL, Jacobs AH, Wyss-Coray T, Vitorica J, Ransohoff RM, Herrup K, Frautschy SA, Finsen B, Brown GC, Verkhratsky A, Yamanaka K, Koistinaho J, Latz E, Halle A, Petzold GC, Town T, Morgan D, Shinohara ML, Perry VH, Holmes C, Bazan NG, Brooks DJ, Hunot S, Joseph B, Deigendesch N, Garaschuk O, Boddeke E, Dinarello CA, Breitner JC, Cole GM, Golenbock DT, Kummer MP. Neuroinflammation in Alzheimer's disease. Lancet Neurology 14(4): 388-405. | 2015 | 2501 |
| 5 | Zlokovic BV. The blood-brain barrier in health and chronic neurodegenerative disorders. Neuron 57(2): 178-201. | 2008 | 2092 |
| 6 | Andersen JK. Oxidative stress in neurodegeneration: cause or consequence? Nature Medicine 10(7): S18-S25. | 2004 | 1376 |
| 7 | Fawcett JW, Asher RA. The glial scar and central nervous system repair. Brain Research Bulletin 49(6): 377-391. | 1999 | 1373 |
| 8 | Dringen R. Metabolism and functions of glutathione in brain. Progress in Neurobiology 62(6): 649-671. | 2000 | 1257 |
| 9 | Ransohoff RM, Perry VH: Microglial Physiology: Unique Stimuli, Specialized Responses, Annual Review of Immunology, 2009: 119-145. | 2009 | 1244 |
| 10 | Hirsch EC, Hunot S. Neuroinflammation in Parkinson's disease: a target for neuroprotection? Lancet Neurology 8(4): 382-397. | 2009 | 1238 |
